# Role of autoimmune hemolytic anemia as an initial indicator for chronic myeloid leukemia: A case report

**DOI:** 10.1097/MD.0000000000019256

**Published:** 2020-02-28

**Authors:** Xiang Li, Sisi Cai, Zhaodong Zhong, Hongxiang Wang, Li Wang, Yong You, Min Zhang

**Affiliations:** a Institution of Hematology, Union Hospital, Tongji Medical College, Huazhong University of Science and Technology, Wuhan; b Institution of Hematology, The central hospital of Wuhan, Tongji Medical College, Huazhong University of Science and Technology, Wuhan, China.

**Keywords:** autoimmune hemolytic anemia, chronic myeloid leukemia, fluorescence in situ hybridization, microarray, prognosis

## Abstract

**Introduction:**

We report here the case of a patient with chronic myeloid leukemia (CML) in the chronic phase who was diagnosed 1 year after receiving a diagnosis of autoimmune hemolytic anemia (AIHA). The objective was to assess if the CML patient progressed from AIHA and explore the underlying factors of the poor outcome after the achievement of molecular complete remission (MCR).

**Patient concerns:**

A patient with AIHA underwent splenectomy because of poor response to immune inhibitors. The spleen biopsy showed reactive hyperplasia.

**Diagnosis:**

The patient was diagnosed with CML because of over-expression of the BCR-ABL (P210) gene in the bone marrow (BM), 1 year after receiving the diagnosis of AIHA.

**Interventions:**

The splenectomy was performed as the patient was unresponsive to the standard treatments consisting of immunoglobulin and dexamethasone. The removed spleen was sent for pathological examination. After she was diagnosed with CML, she received imatinib treatment.

**Outcomes:**

The spleen biopsy confirmed the translocation of 22q11/9q34. No BCR-ABL kinase domain mutation was detected and there was no expression of the WT1 or EVI1 genes. After splenectomy, the number of peripheral white blood cells was consistently higher than normal during the total therapy time for CML even though she showed MCR. Two years after CML was diagnosed, the patient died from severe infection. The BM gene array analysis displayed 3 types of chromosomal abnormalities: gain (14q32.33), uniparental disomy (UPD) Xp11.22-p11.1), and UPD Xp11.1-q13.1.

**Lessons:**

AIHA may be a clinical phase of CML progression in this patient. Both splenectomy and prolonged oral tyrosine kinase inhibitors may have contributed to the high risk of infection and her subsequent death. In addition, the gain of chromosome 14q32.33 may be related to her poor outcome.

## 1 Introduction

Autoimmune hemolytic anemia (AIHA) is caused by autoantibody-induced hemolysis. AIHA is usually idiopathic; however, it may be associated with infection, lymphoproliferative disorders, autoimmune diseases, and several medications. The clinical presentation of AIHA includes anemia with elevated reticulocyte counts in the absence of blood loss, a positive direct antiglobulin (Coombs) test, and spherocyte or red blood cell (RBC) aggregates in the peripheral blood smear.^[1,2]^ Treatment of AIHA is still not evidence based and is mainly accomplished according to expert opinions and individual experience, with few prospective therapeutic trials and large bue dated comprehensive clinical studies.^[3–5]^

Chronic myeloid leukemia (CML) is a hematopoietic disorder characterized by a reciprocal t(9;22)(q34;q11) chromosomal translocation known as the Philadelphia (Ph) chromosome.^[6]^ This abnormal chromosome translocation generates the BCR-ABL oncogene which is necessary (and sufficient) for the transformed phenotype of CML cells.Recently, many genes have been reported in hematological malignancies and play vital roles during clinical therapy. The EVI-1 gene located on the chromosome band 3q26 exhibits several oncogene-like properties and is activated in a subset of most myeloid neoplasms.^[7]^ In acute myeloid leukemia, the expression level of EVI-1 gene has been associated with a poor prognosis, particularly in younger patients.^[8,9]^ In addition, the activation of EVI-1 gene has also been reported in CML blast crisis, though less commonly in the chronic phase (CP).^[10]^ The WT1 gene located on chromosome 11 encodes a zinc finger transcription factor that is involved in cell proliferation and differentiation. Because the expression level of the WT1 gene is correlated with the tumor burden, detection of WT1 gene expression has been used for tracking ofminimal residual disease (MRD), early relapse, or disease progression during and after therapy against myeloid neoplasms, especially acute myeloid leukemia and myelodysplastic syndrome.^[11,12]^ Further, Szanto et al. have reported high WT1 expression levels were seen in all acceleration phase (AP) and blast phase (BP) patients, and the data showed a statistically significant correlation.^[13]^

AIHA may occur during alpha-interferon treatment in patients with CML or other hematological diseases.^[14,15]^ In addition, AIHA is a well-known but relatively rare complication following hematopoietic stem cell transplantation and is reported in approximately 6% of children and 3% of adults.^[16]^ AIHA has not previously been reported as the first symptom of CML. Here we reported an interesting case of a chronic phase CML patient presenting clinical characteristics and BM morphology of typical AIHA initially. After 7 months of tyrosine kinase inhibitors (T K I) therapy, the patient showed a complete molecular and complete hematologic response without WT1 and EVI gene overexpression. However, the patient died from a severe infection 2 years after being diagnosed with CML.

## 2 Case presentation

The study protocol was approved by the Union Hospital Ethics Committee affiliated to the Tongji Medical College, Huazhong University of Science & Technology. The patient had been informed of the course of treatment. The parents of the patient agreed to the publication of this case and provided informed consent. In December 2011, a 16-year-old girl presented with fever, fatigue, and a yellow complexion for a week. Her liver was 2 cm below the costal margin while her spleen was 18.8 × 7.7 cm based on ultrasonography. The hemoglobin level was 39 mg/dL and the reticulocyte count was raised to 15.2%. The white blood cell count was 3.67 × 10^9^/L and the platelet count was 164 × 10^9^/L. The total bilirubin and the direct bilirubin were 186 μmol/L and 26.4 μmol/L respectively. The lactate dehydrogenase level was 524 IU/L. The Direct Coombs test was positive. The BM morphology displayed both myeloid and erythroid hyperplasia. According to Chinese experts’ consensus on the diagnosis and quality of AIHA^[1,2]^, laboratory findings of patients with warm AIHA include a positive direct antiglobulin testing (Coombs test), reticulocytosis, elevated lactate dehydrogenase, elevated indirect bilirubin, and decreased haptoglobin. Considering the patient's clinical manifestations and laboratory tests, the diagnosis was confirmed as AIHA. The patient was treated with intravenous immunoglobulin (2.5 g/kg/d for 5 days), dexamethasone (0.15 mg/kg/d), and antibiotics. In addition, 6 units of washed red blood cells were transfused. Unfortunately, the patient was unresponsive to the standard treatments and hence splenectomy was performed. The spleen histopathology displayed reactive hyperplasia (especially myeloid hyperplasia) induced by chronic hemolytic anemia. The cells were positive for cluster of differentiation (CD)43 and KI67 and a fraction of the cells were positive for CD15 and MPO. The cells were negative for the following antigens: CD2, CD3, CD5, CD7, CD8, CD4, CD20, CD34, CD56, CD68, CD13, CD30, CD117, and TDT.

The patient's platelet count ranged from 1000 × 10^9^/L to 4500 × 10^9^/L during the initial 3 to 6 months post-splenectomy. Hydroxyurea had a limited role in regulating the thrombocytosis. BM aspiration was performed again for persistent thrombocytosis in January 2013, 1 year after the splenectomy. The patient was diagnosed with chronic phase CML based on BM morphology, cytogenetics, and molecular biology. Laboratory tests of bone marrow cells showed a Ph chromosome and a P210 subtype of BCR-ABL. The patient was treated with oral imatinib at a dose of 400 mg/d. We examinedif the AIHA progressed to CMLor if it was a different clinical stage of CML. Fluorescence in situ hybridization test was performed to detect 22q11/9q34 expression in the spleen biopsy according to the manufacturer's instructions (Abbot Molecular, IL). The transcript levels of BCR-ABL, WT1, EVI1, platelet-derived growth factor receptor (PDGFR) rearrangement, JAK2 mutation, and ABL kinase domain mutation were all measured by real-time quantitative polymerase chain reaction. The expression levels were calculated as described in previous studies.^[10–13]^

**Figure 1:**
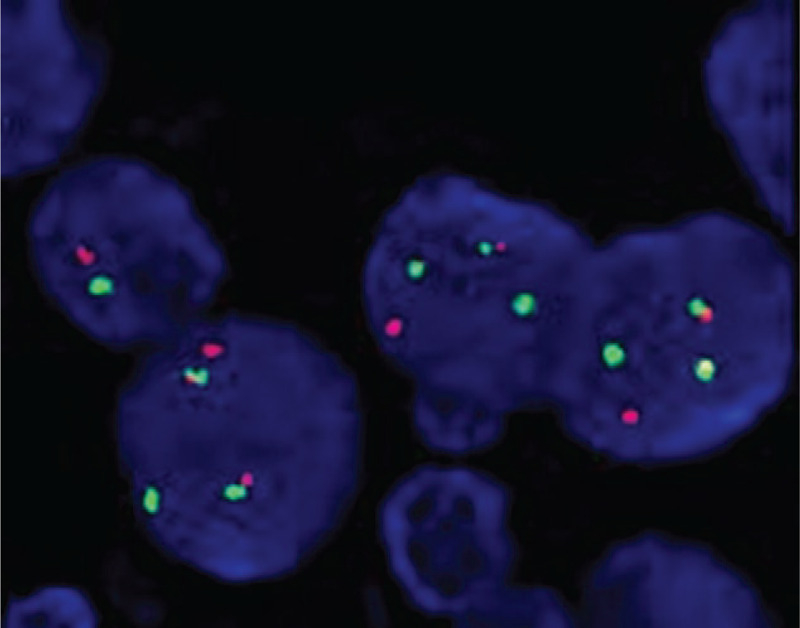
Fluorescent in-situ hybridization (FISH) was performed according to manufacturer instructions by using the BCR/ABL dual color, dual fusion translocation probe. The BCR/ABL probe, when hybridized to a cell containing t (9; 22) (q34;q11), is expected to result in a pattern of a red signal on normal chromosome 9 and a green signal on normal chromosome 22.

Results The data showed a positive expression of 22q11/9q34 in the spleen tissue prior to the presence of clinical or morphological signs of CML (Fig. 1). The patient was diagnosed with chronic phase CML with the Ph chromosome and the P210 subtype of BCR-ABL, 1 year after AIHA was diagnosed. Other related molecular markers such as WT1, EVI1, PDGFR rearrangement, JAK2 mutation, and ABL kinase domain mutation were not detected during therapy. The patient achieved molecular complete remission (MCR) and hematological complete remission (HCR) with normal karyotype 7 months after initiating imatinib therapy. During subsequent target therapy the BM morphology, molecular biology, and cytogenetics were examined. No obvious side effects were identified during imatinib treatment after 3 months.

**Figure 2:**
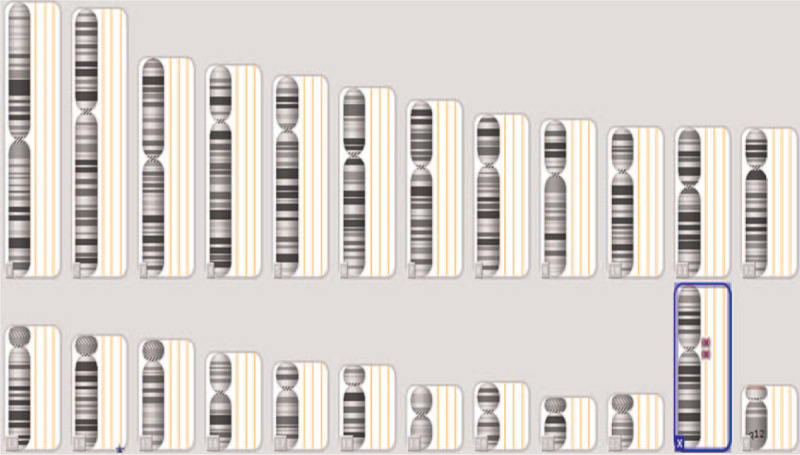
Gene microarray was examined in bone marrow of this case. Gain of 14q32.33 was detected; it includes 642 kilobase (kb) with 3 copy number states.

**Figure 3:**
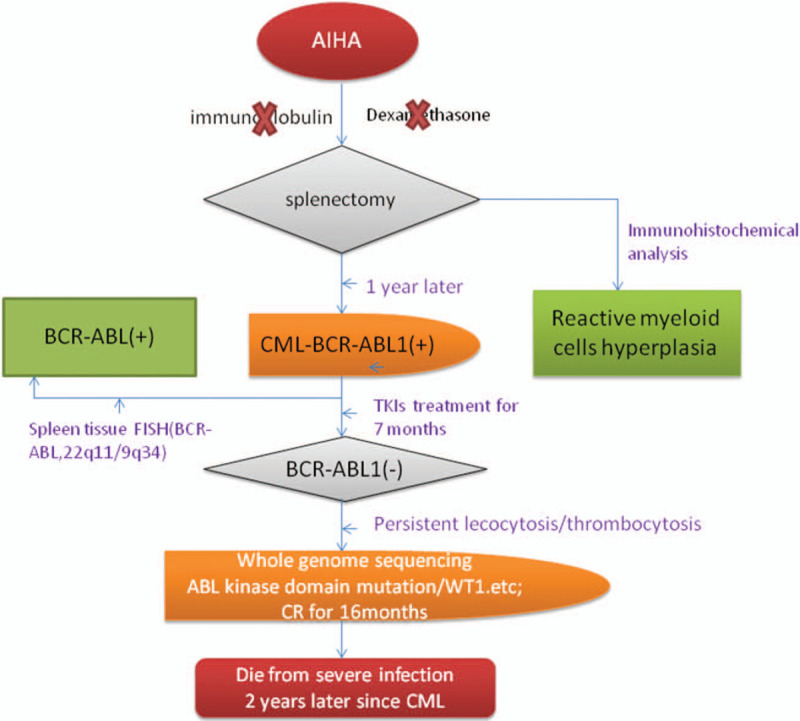
The diagnosis and treatment process for this patient. firstly, the spleen histopathology was displayed as reactive hyperplasia. One year later, the CML was diagnosed based on BM morphology, cytogenetics, and molecular biology. The spleen biopsy was explored again and 22q11/9q34 expression was detected this time. BM = bone marrow. CML = chronic myeloid leukemia. CML = chronic myeloid leukemia.

Although this patient had persistent MCR without any abnormal symptoms, her peripheral white blood cell count was elevated and ranged from 10 × 10^9^/L to 20 × 10^9^/L and the platelet counts fluctuated between 150 × 10^9^/L and 450 × 10^9^/L, accompanied by mild anemia. Furthermore, a BM gene array analysis was performed in December 2014 (approximately 2 years after imatinib treatment) to explore the cause of her excessive peripheral white blood cells and platelet counts. The analysis identified 3 types of chromosomal abnormalities: gain (14q32.33), uniparental disomy (UPD) (Xp11.22-p11.1), and UPD (Xp11.1-q13.1) (Fig. 2); no other poor prognostic chromosomal abnormality was found. Unfortunately, the patient died from a severe infection 2 years after the diagnosis of CML. The timeline is shown in Figure 3.

## 3 Discussion

AIHA may be a clinical syndrome in a specific stage of CML in the present case. Although the patient achieved persistent HCR and MCR 7 months after treatment with imatinib, the patient died from a severe infection in the end. Her death may have been due to the following factors: First, the splenectomy made the patient vulnerable to infection and it may have been the main factor that led to infection in this case. Horowitz et al. have reported that more than 50% patients with CML suffer from postoperative complications after splenectomy and the postoperative deaths were due to septic complications.^[17]^ There were discussions in the early 1970 s that splenectomy may delay or prevent blastic metamorphosis in patients with CML which always proved to be fatal. In these studies, the patients with early-stage CML were randomized into 2 groups: splenectomy or no splenectomy ^[18,19]^ ; however, no survival benefit or reduced rate of blast metamorphosis was observed in either of the studies after splenectomy. In our case, the patient died after splenectomy which might prove that splenectomy performed for hematologic malignancies may be a potentially fatal procedure and thus the benefits need be weighed carefully against the risks.

Second, the introduction of BCR-ABL-TKI for the treatment of hematologic malignancies played a significant impact on the patient's outcome. Contingent upon their targeted and off-target activity, therapy-associated infectious complications may occur. It has been reported that TKI therapy can increase the frequency of opportunistic infections by interfering with the T-cell and natural killer cell activation.^[20,21]^ Therefore, both splenectomy and TKI therapy may have contributed to the severe infection and subsequent death of the patient. Further, it has been suggested that the emergence of BCR-ABL kinase domain mutations may be a part of the disease evolution process and monitoring of the emerging mutations can play a vital role in predicting patients with poor prognosis and revising their therapeutic strategy, irrespective of TKI therapy.^[22,23]^ In addition, many hematology related genes have been reported to have a vital significance in the clinic treatment. Therefore, to evaluate the condition of this patient completely, the expression levels of some oncogenes (such as WT1, EVI1, and PDGFR genes) and a few of the mutations (such as JAK2 and ABL kinase domain mutations) were assessed; however, these results were all negative.

Third, microarray analysis identified 3 types of chromosomal abnormalities: gain (14q32.33), UPD (Xp11.22-p11.1), and UPD (Xp11.1-q13.1). The gain of 14q32.33 encodes for the immunoglobulin heavy chain gene and ADAM6 (ADAM metallopeptidase domain 6, a pseudogene). The gain of 14q32.33 is presumed to cause multiple congenital anomalies/intellectual disability syndromes.^[24]^ The gain of 14q32.33 is also associated with poor clinical outcomes for melanoma patients.^[25]^ The gain of 14q32.33 occurs in 66.7% of patients with diffuse large B cell lymphoma (DLBCL).^[26]^ However, it does not affect the clinical outcome or relapse of DLBCL. Conversely, the loss of 14q32.33 is associated with a favorable clinical outcome in gastric cancer patients.^[27]^ There are no reports describing the relationship between gain/loss of 14q32.22 and leukemia. Thus, considering its close correlation with the prognosis of solid tumors, the gain of 14q32.33 may also play a role in the prognosis of CML. The 2 other types of chromosomal abnormalities were UPD Xp11.22-p11.1 and UPD Xp11.1-q13.1. However, there was no reports on the releationship between these chromosomes withdiseases. Therefore, future studies should focus on 14q32.22 chromosomal abnormalities and the encoded oncogenes to determine their role in CML.

Although 22q11/9q34 expression was confirmed in the spleen biopsy, this patient initially exhibited only clinical characteristics and BM morphology of typical AIHA. The CML was diagnosed 1 year after being diagnosed with AIHA in the case. Thus, AIHA may have been the initial clinical phase of the CML pathogenesis. The patient responded well to the imatinib treatment which is a first-generation tyrosine kinase inhibitor. Although persistent MCR and HCR were achieved during the therapy, the peripheral white blood cell count in this patient was higher when compared to other patients who were in molecular complete response but did not undergo splenectomy. Leukocytosis has been reporetd in human studies following splenectomy.^[28]^ Therefore, the excessive peripheral white blood cells count detected in this patient may have been caused by the splenectomy.

## 4 Conclusion

The present case suggests that AIHA may be considered as an initial clinical stage for CML. Further, despite MCR, and HCR being achieved after 7 months of TKI therapy, the microarray analysis revealed 3 types of chromosomal abnormalities which may be indicative of the poor outcome in this case. Recently, the development of parallel sequencing technologies has demonstrated an unquestionable power to discover genetic changes across entire genomes or protein-coding sequences in human cancers. For example, the potential genetic markers have been discovered in predicting response to IM (Imatinib Mesylate) as frontline therapy and susceptibility markers may also be used as panels for individuals prone to CML.^[29]^ Hence, sequencing technology may be used to obtain accurate information and give individualized recommendations to patients in the clinic. Further, these studies will provide assistance in the identification of new biomarkers or therapeutic targets against leukemia in the future.

## Acknowledgment

We would like to thank Editage (www.editage.com) for English language editing.

## Author contributions

**Conceptualization:** Min Zhang

**Data curation:** Sisi Cai, Zhaodong Zhong.

**Investigation:** Li Wang, Hong Xiang Wang.

**Methodology:** Yong You, Xiang Li.

**Writing – original draft:** Xiang Li

**Writing – review & editing:** Min Zhang, Xiang Li, Zhaodong Zhong.
